# Guideline adherence and health outcomes in diabetes mellitus type 2 patients: a cross-sectional study

**DOI:** 10.1186/s12913-014-0669-z

**Published:** 2015-01-22

**Authors:** Sandra F Oude Wesselink, Hester F Lingsma, Paul BM Robben, Johan P Mackenbach

**Affiliations:** Department of Public Health, Erasmus MC University Medical Center Rotterdam, Room Na-2322, P.O. Box 2040, 3000 Rotterdam, CA The Netherlands; Dutch Health Care Inspectorate, Utrecht, The Netherlands; Institute of Health Policy & Management, Erasmus University Rotterdam, Rotterdam, The Netherlands

**Keywords:** Integrated care, Diabetes mellitus, Guideline adherence, Measurement of quality of care, Health outcomes

## Abstract

**Background:**

The complex disease of diabetes mellitus type 2 (T2DM) requires a high standard of quality of care. Clinical practice guidelines define norms for diabetes care that ensure regular monitoring of T2DM patients, including annual diagnostic tests. This study aims to quantify guideline adherence in Dutch general practices providing care to T2DM patients and explores the association between guideline adherence and patients’ health outcomes.

**Methods:**

In this cross-sectional study, we studied 363 T2DM patients in 32 general practices in 2011 and 2012. Guideline adherence was measured by comparing structure and process indicators of care with recommendations in the national diabetes care guideline. Health outcomes included biomedical measures and health behaviours. Data was extracted from medical records. The association between guideline adherence and health outcomes was analysed using hierarchical linear and logistic regression models.

**Results:**

Guideline adherence varied between different recommendations. For example 53% of the practices had a system for collecting patient experience feedback, while 97% had a policy for no-show patients. With regard to process indicators of care, guideline adherence was below 50% for foot, eye and urine albumin examination and high (>85%) for blood pressure, HbA1c and smoking behaviour assessment. Although guideline adherence varied considerably between practices, after adjusting for patient characteristics we found guideline adherence not to be associated with patients’ health outcomes.

**Conclusions:**

Guideline adherence in Dutch general practices offering diabetes care was not optimal. Despite considerable variations between general practices, we found no clear relationship between guideline adherence and health outcomes. More research is needed to better understand the relationship between guideline adherence and health outcomes, specifically for guidelines that are based on limited scientific evidence.

**Electronic supplementary material:**

The online version of this article (doi:10.1186/s12913-014-0669-z) contains supplementary material, which is available to authorized users.

## Background

The complex disease of diabetes mellitus type 2 (T2DM) requires a high standard of quality of care. The objectives of treatment are controlling glycaemia, blood pressure and blood lipid levels, improving lifestyle behaviour and reducing tobacco use [1]. These objectives are expected to lead to a reduction in the burden of diabetes and its complications. However, optimal treatment is not consistently implemented in clinical practice [2], one possible reason being inadequate adherence to clinical practice guidelines. Such guidelines may well help to improve care, because they specify optimal care for care providers and allow adherence to be monitored. Additional benefits from following guidelines include improving health outcomes, empowering patients, improving the quality of clinical decisions, supporting quality improvement activities, increasing efficiency, and identifying areas where there is insufficient evidence to support optimal care [3].

A national guideline on integrated diabetes care in the Netherlands was formulated in 2007 [4]. It was developed by the Dutch Diabetes Federation and therefore includes contributions from patients, practitioners and scientists. As well as aiming to improve outcomes of care and to reduce the costs of managing T2DM, it also contains general information about diabetes and recommendations on the content, organisation and quality of diabetes care. With a focus on integrated care, it contains instructions for the structure of diabetes care and recommends specific assessments and examinations. Because the guideline is only based on consensus, most of its recommendations are not evidence based. The consensus is based on opinions, on other guidelines and on legislation.

The extent to which care professionals adhere to the broad scope of this guideline is unknown. Previous studies have shown that the proportion of patients in which practices conducted annual measurements of HbA1c, blood pressure, LDL-cholesterol and urine albumin ranges between 49% and 86% [2]. However, for other quality-of-care indicators adherence is unknown.

Although following guidelines should theoretically improve health outcomes, evidence from empirical studies is mixed [5-7]. Previous studies relating guideline adherence to health outcomes have only tested a few specific elements of diabetes guidelines and do not provide conclusions about the overall effect of guideline adherence in T2DM patients. We therefore studied a range of structure and process indicators of care that are mentioned in the guideline.

This study aims to quantify guideline adherence in general practices providing care to T2DM patients in the Netherlands and explores the association between guideline adherence and patients’ health outcomes.

## Methods

### Study population

In the Netherlands, care groups are organisations that provide integrated diabetes care to patients in primary care (Additional file 1: Box S1). These groups consist of 3 to 250 general practitioners, which are funded under a bundled payment system [8]. Care groups are similar to accountable care organizations [9]. From the approximately 100 care groups in the Netherlands, we randomly selected 33. Of these, 18 care groups participated and 15 refused participation (response rate 55%) (Additional file 1: Figure S1). The reasons given for refusal were as follows: too busy with providing care to patients (n = 4), no compensation for time loss due to research (n = 2), do not agree with purpose of research (n = 1), currently involved in other research (n = 1) and unknown (n = 7).

Each participating care group selected one or two practices to participate in this study, based on their availability to participate in research. In total 32 practices participated in our study, together employing 32 practice nurses. Practice nurses either have a registered nursing degree or are practice assistants who have followed a two-year practice nursing degree.

Patient and practice data were collected from the general practices cross-sectionally between June 2011 and July 2012. For each practice, based on the schedule of last month, we randomly selected between 7 and 18 patients who had had a check-up in the last month. For these patients, data from the medical records was extracted by the practice nurses, together with the research assistants. In addition, the practice nurses were also asked to complete a questionnaire about guideline adherence.

No-one (care groups, practices or patients) received financial compensation for participating in this study. The local ethics committee of Erasmus University Medical Centre waived ethical approval for this particular analysis. A written informed consent was obtained from all participating practice nurses.

### Study variables and definitions

The main outcome parameters of the study were guideline adherence in the practices and health outcomes in the patients. We also collected data on patient characteristics for use in the statistical analysis.

All variables were obtained from patient files and the most recent measurements were used. Health outcomes were BMI, systolic blood pressure, HbA1c, LDL cholesterol, urine albumin, glomerular filtration rate (GFR) and smoking behaviour. BMI (kg/m2), blood pressure (mmHg) and smoking behaviour (yes/no) were assessed in general practices and documented in patient files. Glucose (mmol/L), HbA1c (mmol/mol), LDL cholesterol (mmol/L), urine albumin (mg/L) and GFR (ml/min) were assessed in cooperation with diagnostic centres and documented in patient files. Measurements from before 2009 were not used, because these health outcomes were regarded as potentially outdated.

Guideline adherence was assessed by considering both structure and process indicators of care. Structure indicators, defined at practice level, were assessed by asking practice nurses whether they: have a system for collecting patient experience feedback, have regulations on access to patient files, have quarterly multidisciplinary meetings, have policies for checking medical equipment, have received training in self-management and have policies for no-show patients. Each question was given a numerical score of 1 (yes) or 0 (no) and these numbers were added to compose an aggregated score for structure indicators of care at each practice, ranging from 0 for the lowest to 6 for the highest quality of care in terms of structures (Table 1).

**Table 1:** **Construction of guideline adherence scores, divided into structures and process indicators of care**

**Structure score** ^**1**^	**Points**
System for collecting patient experience feedback	0 or 1
Regulations on access to patient files	0 or 1
Quarterly multidisciplinary meetings	0 or 1
Policies for checking medical equipment	0 or 1
Practice nurse trained in self-management	0 or 1
Policy for no-show patients	0 or 1
*Score range*	*0-6*
**Process score** ^**2**^	
Annual assessment of BMI	0-1
Annual assessment of blood pressure	0-1
Annual assessment of HbA1c	0-1
Annual assessment of LDL cholesterol	0-1
Annual assessment of urine albumin	0-1
Annual assessment of GFR	0-1
Annual assessment of smoking behavior	0-1
Annual foot examination	0-1
Annual eye examination	0-1
*Score range*	*0-9*

^1^Structure score, composite score of different structure indicators of care, scoring: present = 1 point, absent = 0 points.

^2^Process score, composite score of different process indicators of care, the proportion of patients per practice that were tested annually for each indicator, range between 0 and 1.

We defined process indicators at the patient level as being the previously mentioned annually measured health outcomes and obtained data from the patient records. A measurement was considered to be annual if the time period between two measurements was less than 366 days. For each practice we then calculated the proportion of patients in whom annual measurements had been done, as recommended by the guidelines. Once more an aggregated score for processes of care at each practice was composed, ranging from 0 for the lowest to 9 for the highest quality of care in terms of processes (Table 1).

For each practice, we analysed guideline adherence according to structure and process indicators of care. Each structure indicator was coded as yes/no per practice, resulting in an aggregated percentage of guideline adherence in all practices for each structure indicator. For process indicators we also calculated an aggregated percentage of patients per practice, resulting in an average guideline adherence across all practices for each process indicator.

The patient characteristics consisted of demographic factors and clinical factors. Demographic factors were age at data collection (years), sex and an indicator of socio-economic status (SES). Clinical factors were years since diagnosis of T2DM (between diagnosis and the moment of data collection) and comorbidities (defined using ICPC codes) [10]. Comorbidities unrelated to T2DM were based on general national guidelines [11] while comorbidities related to T2DM were derived from the National Diabetes Guideline (Additional file 1: Table S2) [4]. All patient characteristics, except SES, were collected from patient files. The SES was based on the neighbourhood (postal code) of the general practice where the patient was treated. This score was obtained from a government agency (Netherlands Institute for Social Research) [12] and ranges from -10 to +10. A higher score represents a higher SES.

### Statistical analysis

Guideline adherence was described at the practice level. Patient characteristics and health outcomes were described at both the patient and practice levels. The associations were evaluated using hierarchical linear and logistic regression models. In hierarchical models, the clustering of patients within practices is taken into account [13].

Variation in guideline adherence between practices was described per indicator with interquartile ranges (IQR). The statistical significance of the variation between practices was tested in hierarchical regression models with the structure or process indicator of care as the dependent variable and a random intercept for the practices. Variation in health outcomes between practices was analysed with hierarchical regression models with the health outcome as the dependent variable and a random intercept for the practices.

Finally, to analyse the association between guideline adherence and health outcomes, adjusted for patient characteristics, we added to the model the aggregated score for structures or processes per practice as independent variable. The different health outcomes served as dependent variable. General practice was included in the model as a random intercept. Each health outcome was analysed separately and patients with missing outcomes (9% at most) were excluded from the analysis. Guideline adherence was analysed at practice level to avoid confounding by indication. Confounding by indication is a common problem in observational studies, where treatment is usually only given to patients who require it [14]. Without adjustment on practice level in the data analysis, appropriate treatment will always be related to poor health outcome, particularly in individual-level studies. By analysing adherence at the practice level, we can test whether practices with generally good guideline adherence have good outcomes.

The one covariate for which values were missing in about 50% of cases, namely year since diagnosis, was imputed with linear regression analysis based on seven covariates (sex, age, related and unrelated comorbidities, SES, structure and process quality scores).

From the regression models we derived beta scores (in the case of a continuous outcome) or odds ratios (in case of a binary outcome) and 95% confidence intervals. For regression analyses we used statistical software package SAS version 9.3 (PROC MIXED and PROC GLIMMIX) (SAS Institute Inc., Cary, NC) and for other analyses SPSS version 21.0 (IBM Inc., Somers, NY).

## Results

Thirty-two general practices participated in the study. We included between 7 and 18 patients per practice, resulting in 363 patients in total. Guideline adherence was assessed according to structure and process indicators of care (Table 2). Structure scores are expressed as the proportion of practices that answered positively to each of the questions about guideline adherence. For example, only 53% of practices reported that they systematically collect patient experience feedback. Adherence was also below 70% for regulations on access to patient files (63%) and for conducting quarterly multidisciplinary meetings (66%). For other structural aspects, guideline adherence was better: almost all practices had a policy for no-show patients (97%), a practice nurse trained in self-management (94%) and policies for checking medical equipment (91%). Overall, there was limited variation between practices for guideline adherences according to structure indicators of care; the IQR of the total structure score was 4 to 5 (p = 1.00).

**Table 2:** **Description of mean scores for guideline adherence indicators in general practices (n = 32)**

**Structure score**	**Guideline adherence (proportion of practices)**	**IQR**	p-value*
System for collecting patient experience feedback	0.53		
Regulations on access to patient files	0.63		
Quarterly multidisciplinary meetings	0.66		
Policies for checking medical equipment	0.91		
Practice nurse trained in self-management	0.94		
Policy for no-show patients	0.97		
*Total structures of care score (points)*	*mean: 4.7 (scale 0-6)*	*4 - 5*	*1.00*
**Process score**	**Guideline adherence (proportion of patients per practice)**	**IQR**	p-value*
Annual assessment of BMI	0.70	0.41 - 1.00	0.00
Annual assessment of blood pressure	0.97	0.92 - 1.00	1.00
Annual assessment of HbA1c	0.91	0.85 - 1.00	0.00
Annual assessment of LDL cholesterol	0.59	0.42 - 0.76	0.00
Annual assessment of urine albumin	0.49	0.35 - 0.66	0.01
Annual assessment of GFR	0.65	0.48 - 0.78	0.00
Annual assessment of smoking behavior	0.89	0.86 - 0.98	0.00
Annual foot examination	0.33	0.12 - 0.49	0.00
Annual eye examination	0.28	0.10 - 0.43	0.03
*Total processes of care score (points)*	*mean: 5.9 (scale 0-9)*	*5.3 - 6.6*	*1.00*

*Differences between practices, tested in hierarchical regression model, without other independent factors.

IQR, interquartile range; BMI, body mass index; HbA1c, glycosylated haemoglobin; LDL cholesterol, low-density lipoprotein cholesterol; GFR, glomerular filtration rate.

Guideline adherence measured according to process indicators of care, also shown in Table 2, is expressed as the proportion of patients receiving treatment according to the guideline. For example, 70% of patients were assessed annually for BMI and the IQR for the percentages per practice was 41% to 100%. Guideline adherence was below 50% for the assessment of urine albumin (49%) and for the examination of the feet (33%) and eyes (28%). However, guideline adherence was above 85% for blood pressure (97%), HbA1c (90%) and smoking behaviour (89%). The scores for the process indicators varied largely between practices: the IQR was often more than 30%. The IQR of the total process score was 5.3-6.6 (p = 1.00).

Table 3 shows the patient characteristics and health outcomes. Median age of the 363 patients was 65 years and about half were males (49%). Median HbA1c was 50 mmol/mol and median GFR was 68 ml/min. The percentage of missing health outcomes was low; less than 7%. The study population was comparable to those of previous studies [7,15].

**Table 3:** **Description of patient characteristics and unadjusted health outcomes for all patients (n = 363) and at practice level (n = 32)**

		**Patients**			**Practices**		
		**n**	**Median**	**IQR**	**Median**	**IQR**	p-value*
**Patient characteristics**							
Age (years)		363	65	58 - 74	66	63 - 70	0.02
Sex (% males)		363	49%		52%	43 - 58%	1.00
Years since diagnosis		175	6	3 - 9	7	5 - 9	0.04
Number of related comorbidities^1^		363	1	0 - 1	0.6	0.4 - 0.9	0.00
Number of unrelated comorbidities^2^		363	0	0 - 1	0.4	0.2 - 0.5	0.02
SES indicator^3^	*Low*	363	46%		44%		
	*Middle*		34%		34%		
	*High*		20%		22%		
**Health outcomes**							
BMI (kg/m^2^)^4^		343	29	26 - 33	29	29 - 31	0.10
Systolic blood pressure (mmHg)^5^		363	135	123 - 145	134	131 - 140	0.02
HbA1C (mmol/mol)^6^		361	50	45 - 56	51	48 - 55	0.01
LDL cholesterol (mmol/L)^7^		358	2.3	1.9 - 2.9	2.5	2.2 - 2.6	1.00
Urine albumin (mg/L)^8^		341	6	3 - 13	11	6 - 32	0.06
GFR (ml/min)^9^		356	68	60 - 89	74	63 - 86	0.00
Smoking (% smokers)		353	18%		17%	11 - 25%	1.00

*Differences between practices, tested in hierarchical regression model, without other independent factors.

ICC of patient characteristics, range: 0.07-0.16. ICC of outcomes, range: 0.04-0.25.

^1^in total 12 ICPC codes.

^2^in total 63 ICPC codes.

^3^Score calculated nationally based on the postal code of the general practice where the patient was treated, low = lowest tertile, middle = middle tertile, high = highest tertile.

^4^Body mass index (range in this study: 19-53).

^5^Systolic blood pressure (range in this study: 95-197).

^6^Glycosylated haemoglobin (range in this study: 32-99).

^7^Low-density lipoprotein cholesterol (range in this study: 0.5-5.9).

^8^Urine albumin (range in this study: 0-1023).

^9^Glomerular filtration rate, higher is better (range in this study: 13-178).

IQR, interquartile range.

Several patient characteristics varied significantly between practices. For example mean age per practice had an IQR of 63 to 70 years (p = 0.02). Health outcomes also differed across practices. For example, the IQR of HbA1c was 48 to 55 mmol/mol (p = 0.01) and for GFR IQR was 63 to 86 ml/min (p = 0.00) (Table 3). The patient characteristics were not associated with guideline adherence, indicating that adherence was not better or worse in specific patient groups (data not shown).

When we explored our patient population further using regression models, we found several associations between patient characteristics and health outcomes (Additional file 1: Table S1). For example, HbA1c was higher in patients who had a longer duration of diabetes (95% CI 0.0-0.4) or a higher number of related comorbidities (95% CI 0.2-3.2). Low GFR was associated with a higher age, meaning that kidney function worsens with older age (95% CI -0.9;-0.6).

Finally, we explored the associations between guideline adherence and health outcomes (Table 4). We found no clear relationships between guideline adherence and the health outcomes under study. The estimated effects point to both positive and negative associations between guideline adherence and health outcomes. Only systolic blood pressure was positively related to both structure and process indicators of care, but this relationship was not statistically significant.

**Table 4:** **Associations between guideline adherence and health outcomes, analysed with hierarchical linear and logistic regression models (n = 363)**

	**β**	**(95% CI)**
**BMI** (n = 343)		
Structure	−0.03	(-0.88;0.82)
Process	0.51	(-0.24;1.26)
**Systolic blood pressure** (n = 363)		
Structure	1.70	(-1.08;4.47)
Process	1.97	(-0.46;4.39)
**HbA1C** (n = 361)		
Structure	0.75	(-1.01;2.50)
Process	−0.64	(-2.19;0.90)
**LDL cholesterol** (n = 358)		
Structure	−0.017	(-0.120;0.086)
Process	−0.013	(-0.107;0.080)
**Urine albumin** (n = 341)		
Structure	−1.58	(-16.44;13.29)
Process	3.32	(-10.20;16.84)
**GFR** (n = 356)		
Structure	4.54	(-0.39;9.47)
Process	−2.53	(-6.99;1.94)
Smoking* (n = 353)		
Structure (OR)	1.13	(0.80;1.60)
Process (OR)	0.93	(0.68;1.28)

*Analysed with logistic regression: estimated odds ratio (OR) (smoker = 1, non-smoker = 0).

CI, confidence interval; BMI, Body Mass Index (kg/m2); Systolic blood pressure (mmHg); HbA1c, Glycosylated haemoglobin (mmol/mol); LDL cholesterol, Low-density lipoprotein cholesterol (mmol/L); Urine in albumin (mg/L); GFR, Glomerular Filtration Rate (higher is better) (ml/min).

Structure: structures of care score per practice, see Table 1.

Process: processes of care score per practice, see Table 1.

All models control for age, sex, years since diagnosis, number of related and unrelated comorbidities and social economic status.

## Discussion

### Summary of main findings

Guideline adherence varied between different recommendations. For example 53% of the practices had a system for collecting patient experience feedback, while 97% had a policy for no-show patients. With regard to process indicators of care, guideline adherence was below 50% for foot, eye and urine albumin examination and high (>85%) for blood pressure, HbA1c and smoking behaviour assessment. Although guideline adherence varied considerably between practices, after adjusting for patient characteristics we found guideline adherence not to be associated with patients’ health outcomes.

### Strengths and limitations

The study has several strengths and limitations. One strength is that we retrieved actual guideline adherence with regard to several indicators at the patient level, which is more precise than information collected at the practice level. A second strength is the method of data collection, as the research assistant collected the data from the patient files together with the practice nurse, our data were reliable with only a low number of missing values.

A limitation of this study was the substantial number of care groups that refused to participate. Of the 33 care groups that were approached, 15 refused to participate. Since it is possible that refusal is associated with poor guideline adherence, actual guideline adherence may be worse than that found in this study.

Another limitation of this study was the cross-sectional design, with guideline adherence and health outcomes being measured at the same time. As in practice it will take some time before better guideline adherence results in improved health outcomes, we have to assume that differences in guideline adherence between practices are relatively constant over time. Violation of this assumption, i.e. rapid changes in guideline adherence over time in individual practices, may have led to an underestimation of associations between guideline adherence and health outcomes.

Two arbitrary choices were made during data analysis, that may also have affected our results: firstly, we constructed the guideline adherence scores based on a comparable scientific paper [5], and secondly, for the process indicators we used the number of days between check-ups to determine whether or not there was adherence to the guideline. In order to assess the robustness of the different choices made regarding these two issues, we performed a series of sensitivity analyses in which the scoring was adjusted as follows: (1) putting double weight on multidisciplinary meetings, policy for no-show patients and training in self-management, as these are directly related to patient care; (2) putting double weight on all indicators with the exception of the foot and eye examination; (3) giving three points instead of one for annual assessment of the specific outcome under study; (4) increasing the number of days between assessments considered to be as adherent from 366 to 400, 450 or 500 days. All four sensitivity analyses yielded the same results as those found in the main analyses (data not shown).

Finally, when comparing our results with those of others, it should be taken into account that hard morbidity and mortality outcomes are frequently considered to be more important and more relevant to patients than the health outcomes used in this study. However, these endpoints, that include cardiovascular events and death, occur too infrequently in a general practice population [16] to be used as an outcome in our study.

A further consideration is that while the general practices were selected by the care groups, we do not expect selection bias to have occurred as the care groups were not aware of the aim of this study in advance. Furthermore, patients were selected at random.

### Interpretation

Guideline adherence was suboptimal for several structure and process indicators of care. With regard to structure, a system for collecting patient experience feedback had not yet been widely implemented, although some practices had started a first investigation. Our finding that regulations on access to patient files also scored below 70% was likely due to the fact that such agreements often do not cover the care group as a whole, as stated by the practices nurses. Practices nurses reported also that quarterly multidisciplinary meetings did not cover all care providers of the care groups and that these meetings sometimes did not include discussion of individual patients.

With regard to process, guideline adherence was limited for half of the process indicators of care, with three scoring below 50%: assessment of urine albumin and examination of the eyes and feet. Eye examinations were always conducted outside the general practice and the practice did not always receive the patient’s report after these examinations, as stated by practice nurses. In contrast, foot examinations were performed in the general practice, but often less than once a year. The fact that assessment of urine albumin scored lower than other laboratory assessments may well be due to patients not always supplying urine samples to the laboratory when necessary.

Since many quality indicators scored below 70% adherence, we conclude that guideline adherence is suboptimal in general practices offering diabetes care. Not only are our results in line with those of previous research [2], but they also give insight into guideline adherence with respect to structure indicators of care. The fact that we found large differences in guideline adherence between practices is also consistent with previous studies in diabetes care [17].

If we assume that guidelines describe optimal and evidence-based care, then variation in guideline adherence is undesirable. However, we found no relationship between guideline adherence and health outcomes. This finding is in line with those of other studies. For example, Ackerman et al found that in diabetes patients improvements in processes of diabetes care were not associated with improvements in health outcomes [18]. Another study showed that improved processes of care were associated with an improved mental health score, but not with a physical health score [5]. A systematic review also concluded that structure and process indicators of diabetes care are largely unrelated to surrogate and hard outcomes [19]. While these previous studies tested just a few specific elements of diabetes guidelines, our results expand on these previous results and suggest that guideline adherence in general is not associated with health outcomes.

The guideline that we studied is not completely evidence based. While it is comparable to the NICE diabetes guidelines [20], not every single element of the guideline has been underpinned with evidence [21]. This absence of evidence underlying some aspects of the guideline might be the explanation for the findings in our study – and in most previous studies – that adherence to elements of diabetes guidelines appears to have no effect on health outcomes. This is supported by the fact that, in other disease fields where the guidelines are more evidence-based, an association between adherence and health outcomes has been found [22-24]. In the case of T2DM, the lack of evidence for single elements of the diabetes guideline is at least partly due to the heterogeneity of the patient population, which implies that not all guideline recommendations apply to all patients. Additionally, while most recommendations concern the frequency with which a measurement should be done, e.g. measuring albumin once a year, they do not specify the actions that should subsequently be taken to improve outcome. However, the lack of a relationship between adherence and health outcomes might also partly be explained by the degree of self-management in diabetes: because it is a lifestyle-related disease, the role played by the patient is central. If the patient does not adapt his or her lifestyle to the disease, then his or her health will not improve [25]. Nevertheless, the lack of evidence underpinning clinical practice guidelines is a common problem in many disease fields [26] and cannot be ruled out as an explanation for our results.

The weak scientific evidence underpinning the guideline might also explain the relatively poor adherence: previous research has shown that lack of familiarity and lack of outcome expectancy can sometimes be barriers to guideline adherence [27]. Another possible explanation for our results is that Dutch general practices might use alternative guidelines [28]. Nevertheless, as the guideline we studies is the only guideline for integrated diabetes care in the Netherlands, it was this guideline that the Dutch Healthcare Inspectorate used to evaluate integrated diabetes care in 2011 [29]. The Dutch Diabetes Federation, who developed the guideline, represents a broad spectrum of stakeholders and the guideline therefore is acknowledged by all professions involved in diabetes treatment.

## Conclusion

In conclusion, guideline adherence in Dutch general practices offering diabetes care was not optimal. Despite considerable variations between general practices, we found no clear relationship between guideline adherence and health outcomes. For clinical practice, policy making and supervision it is important to consider the large variation in guideline adherence. While quality improvement initiatives might reduce the observed variation, our study suggests that better guideline adherence will not automatically lead to better health outcomes.

More research is needed to better understand the relationship between guideline adherence and health outcomes, specifically for guidelines that are based on limited scientific evidence.

## Additional file

Additional file 1: Table S1. Associations between patient characteristics and health outcomes, analysed with hierarchical linear and logistic regression models (n = 363). **Table S2.** List of comorbidities related and unrelated to diabetes. **Box S1.** Care groups. **Figure S1.** Flow chart of participating care groups [30].
