# Supplementary material for: Guideline adherence and health outcomes in diabetes mellitus type 2 patients: a cross-sectional study
Source: BMC Health Serv Res. 2015 Jan 22;15:22. doi: 10.1186/s12913-014-0669-z (PMC4312465; doi:10.1186/s12913-014-0669-z)
Supplement: Additional file 1: Table S1. — Associations between patient characteristics and health outcomes, analysed with hierarchical linear and logistic regression models (n = 363). Table S2. List of comorbidities related and unrelated to diabetes. Box S1. Care groups. Figure S1. Flow chart of participating care groups [30]. [file 12913_2014_669_MOESM1_ESM.docx]

**Table S1 Associations between patient characteristics and health outcomes, analysed with hierarchical linear and logistic regression models (n=363)**

|  | **β** |  | **(95% CI)** |
| --- | --- | --- | --- |
| **BMI** |  |  |  |
| Age (years) | -0.10 |  | (-0.16;-0.05) |
| Sex (0=female, 1=male) | -0.61 |  | (-1.65;0.43) |
| Years since diagnosis | -0.05 |  | (-0.16;0.06) |
| Number of related comorbidities | 0.84 |  | (0.07;1.62) |
| Number of unrelated comorbidities | 0.86 |  | (0.02;1.69) |
| SES indicator (per practice) | 0.41 |  | (-0.21;1.03) |
| **Systolic blood pressure** |  |  |  |
| Age (years) | 0.20 |  | (0.04;0.36) |
| Sex (0=female, 1=male) | 1.47 |  | (-1.78;4.72) |
| Years since diagnosis | -0.16 |  | (-0.51;0.18) |
| Number of related comorbidities | 5.86 |  | (3.40;8.32) |
| Number of unrelated comorbidities | -0.71 |  | (-3.29;1.87) |
| SES indicator (per practice) | 1.10 |  | (-0.95;3.14) |
| **HbA1c** |  |  |  |
| Age (years) | -0.03 |  | (-0.13;0.06) |
| Sex (0=female, 1=male) | -0.05 |  | (-1.97;1.86) |
| Years since diagnosis | 0.22 |  | (0.02;0.42) |
| Number of related comorbidities | 1.70 |  | (0.24;3.16) |
| Number of unrelated comorbidities | 0.37 |  | (-1.16;1.91) |
| SES indicator (per practice) | -0.16 |  | (-1.42;1.11) |
| **LDL cholesterol** |  |  |  |
| Age (years) | -0.012 |  | (-0.020;-0.004) |
| Sex (0=female, 1=male) | -0.270 |  | (-0.438;-0.102) |
| Years since diagnosis | 0.001 |  | (-0.016;0.019) |
| Number of related comorbidities | -0.051 |  | (-0.172;0.071) |
| Number of unrelated comorbidities | 0.106 |  | (-0.024;0.236) |
| SES indicator (per practice) | -0.033 |  | (-0.110;0.043) |
| **Urine albumin** |  |  |  |
| Age (years) | -0.60 |  | (-1.53;0.33) |
| Sex (0=female, 1=male) | 8.64 |  | (-9.86;27.15) |
| Years since diagnosis | 0.51 |  | (-1.44;2.46) |
| Number of related comorbidities | 17.27 |  | (2.90;31.65) |
| Number of unrelated comorbidities | 7.25 |  | (-7.39;21.88) |
| SES indicator (per practice) | -1.62 |  | (-12.40;9.16) |
| **GFR** |  |  |  |
| Age (years) | -0.75 |  | (-0.94;-0.55) |
| Sex (0=female, 1=male) | 0.97 |  | (-2.79;4.73) |
| Years since diagnosis | 0.19 |  | (-0.21;0.59) |
| Number of related comorbidities | -0.45 |  | (-3.41;2.51) |
| Number of unrelated comorbidities | 0.03 |  | (-3.04;3.10) |
| SES indicator (per practice) | 0.22 |  | (-3.45;3.89) |
| **Smoking*** |  |  |  |
| Age (years) (OR) | 0.99 |  | (0.97;1.02) |
| Sex (0=female, 1=male) (OR) | 2.09 |  | (1.18;3.70) |
| Years since diagnosis (OR) | 0.93 |  | (0.87;1.00) |
| Number of related comorbidities (OR) | 1.24 |  | (0.85;1.81) |
| Number of unrelated comorbidities (OR) | 1.57 |  | (1.05;2.35) |
| SES indicator (per practice) (OR) | 0.95 |  | (0.74;1.22) |

*Analysed with logistic regression: estimated odds ratio (OR) (smoker=1, non-smoker=0)

CI, confidence interval; BMI, body mass index; HbA1c, glycosylated haemoglobin; LDL cholesterol, low-density lipoprotein cholesterol; GFR, glomerular filtration rate (higher is better);

**Table S2 List of comorbidities related and unrelated to diabetes**

|  | **ICPC code** |
| --- | --- |
| **Related comorbidities** |  |
| Angina pectoris | K74 |
| Acute myocardial infarction | K75 |
| Hypertension | K86 and K87 |
| Transient ischemic attack | K89 |
| Stroke | K90 |
| Intermittent claudication | K92 |
| Aneurysm aortae | K99 |
| Diabetic neuropathy | N92 |
| Depression | P03 and P76 |
| **Unrelated comorbidities** |  |
| Tuberculosis | A70 |
| HIV/AIDS | B90 |
| Cancer | A79, B72, B73, D74, D75, D76, D77, L71, |
|  | N74, R84, R85, S77, T71, U75, U76, U77, |
|  | W72, X75, X76, X77, Y77, Y78 |
| Peptic ulcer | D85, D86 |
| Ulcerative colitis | D94 |
| Visual disturbance | F83, F84, F92, F93, F94 |
| Hearing impairment | H84, H85, H85 |
| Congenital heart defect | K73 |
| Heart failure | K77 |
| Chronic neck and back problems | L83, L84, L85, L86 |
| Rheumatoid arthritis | L88 |
| Osteoarthritis | L89, L90, L91 |
| Osteoporosis | L95 |
| Congenital neurological disorder | N85 |
| Multiple sclerosis | N86 |
| Parkinson’s disease | N87 |
| Epilepsy | N88 |
| Chronic alcohol abuse | P15 |
| Dementia | P70 |
| Schizophrenia | P72 |
| Anxiety disorder, other neurosis, PTSS | P74, P79 |
| Anorexia nervosa | T06 |
| Mental retardation | P85 |
| COPD | R91, R95 |
| Asthma | R96 |
| Eczema | S87, S88 |

# **Box S1** Care groups

In the Netherlands, care groups are organisations that provide integrated diabetes care to patients in primary care. Care groups consist of 3 to 250 general practitioners, which are funded under a bundled payment system [[8](#_ENREF_8)]. Bundled payment means that health insurance companies pay a single fee for all medical services involved in an episode of care [[30](#_ENREF_30)]; in this case, paying a yearly fee for each diabetes patient in the care group. Care groups are the main contractor of a diabetes care program, and are responsible for the organisation, coordination and delivery of diabetes care.

Care groups consist of multiple health care providers and are often owned by general practitioners. Both general practitioners and practice nurses provide diabetes care within the care group, with practice nurses mainly performing check-ups for diabetes patients. Other care providers are contracted by the care group.

While care groups are similar to accountable care organisations [[9](#_ENREF_9)], accountable care organisations in the United States have a much broader scope, which includes hospital care. Legal requirements for care groups are far more extensive in the U.S. than in the Netherlands.

**Figure S1 Flow chart of participating care groups**
